# DNA methylation signature of cognitive reserve moderates CSF tau pathology in prodromal Alzheimer’s disease

**DOI:** 10.21203/rs.3.rs-8369919/v1

**Published:** 2026-01-06

**Authors:** David Lukacsovich, Juan I. Young, Lissette Gomez, Brian W. Kunkle, Zhixin Mao, Wei Zhang, X. Steven Chen, Deirdre M. O’Shea, Tatjana Rundek, Eden R. Martin, Lily Wang

**Affiliations:** 1 Division of Biostatistics, Department of Public Health Sciences, University of Miami, Miller School of Medicine, Miami, FL 33136, USA; 2 Dr. John T Macdonald Foundation Department of Human Genetics, University of Miami, Miller School of Medicine, Miami, FL 33136, USA; 3 John P. Hussman Institute for Human Genomics, University of Miami Miller School of Medicine, Miami, FL 33136, USA; 4 Department of Neurology, University of Miami Miller School of Medicine, Miami, FL 33433, USA; 5 Evelyn F. McKnight Brain Institute, University of Miami School of Medicine, Miami, FL 33136, USA; 6 Sylvester Comprehensive Cancer Center, University of Miami, Miller School of Medicine, Miami, FL 33136, USA

**Keywords:** cognitive reserve, DNA methylation, CSF pTau181, memory

## Abstract

**Background:**

Cognitive reserve (CR) refers to differences in the adaptability of cognitive processes that modify the impact of Alzheimer’s disease (AD) pathology on cognitive performance. Currently there are no established blood-based biomarkers of CR in prodromal AD. In this study, we operationalize CR as memory reserve, defined as moderation (attenuation) of the CSF pTau181-memory association. DNA methylation (DNAm) integrates genetic and environmental influences and may capture biological processes that mitigate the impact of AD pathology on memory. We aimed to identify blood DNAm loci that moderate the association between cerebrospinal fluid (CSF) phosphorylated tau (pTau181) and memory in mild cognitive impairment (MCI). We also sought to determine if a DNAm-based signature of memory reserve predicts future memory decline.

**Methods:**

We analyzed 92 amyloid positive MCI participants from the Alzheimer’s Disease Neuroimaging Initiative (ADNI) with blood DNAm, CSF pTau181, and memory scores (PHC_MEM) collected at the same visit. We first regressed memory scores on covariates (age, sex, number of *APOE4* alleles, estimated major immune cell type proportions) and used the residuals as covariate-adjusted memory scores. At each CpG, we then fitted linear models of memory on DNAm, pTau181, and their interaction. Inflations were corrected using the bacon method. We identified differentially methylated regions (DMRs), assessed pathway enrichment, and performed integrative analyses incorporating external resources including expression quantitative trait methylation (eQTM), methylation quantitative trait loci (mQTL) databases, AD genome-wide association study summary statistics, and blood-brain DNAm correlations. A methylation score was constructed and evaluated in linear mixed-effects models of longitudinal memory in 88 participants with follow-up information.

**Results:**

After removing CpGs with low variability, we identified 6 CpGs with suggestive significance for DNAm×pTau181 interaction (*P*-value < 1× 10^−5^) and 11 DMRs that passed multiple comparisons correction. These loci mapped to genes involved in synaptic function, vascular and blood-brain barrier integrity, amyloid clearance, immune and metabolic regulation. Almost all showed no strong marginal associations with pTau181 or memory, supporting a moderating rather than mediating role. Pathway analysis revealed enrichment of adipocytokine signaling and adipose metabolic pathways, and a number of CpGs associated with mQTLs overlapped with AD genetic risk loci. A higher baseline MRS attenuated the pTau-memory association and significantly associated with slower future memory decline, independent of age, sex, education, *APOE* ε4, and baseline pTau181.

**Conclusions:**

Blood DNAm patterns that moderate the pTau-memory relationship capture biology underlying memory reserve involving synaptic, vascular, immune, and metabolic pathways, and can be summarized into an MRS that predicts longitudinal memory trajectories in MCI. These findings support blood DNAm as a promising, non-invasive biomarker of cognitive resilience to AD pathology.

## BACKGROUND

Alzheimer’s disease (AD) is a major public health challenge and one of the most financially costly diseases^1^. The pathological hallmark of AD is the accumulation of aggregated amyloid beta plaques and tau neurofibrillary tangles in the brain. However, there is considerable heterogeneity in clinical presentation that cannot be fully explained by the extent of AD pathology. Autopsy studies revealed that about one-quarter of cognitively normal older adults have sufficient amyloid pathology in the brain to meet neuropathological criteria for AD.^2,3^ It has been estimated that age-related neuropathological burden explains only about half of the variance in cognitive decline^4–6^, suggesting that additional factors are involved in the clinical manifestation of AD.

Cognitive reserve (CR) refers to differences in the adaptability of cognitive processes that modify the impact of AD pathology on cognitive performance^7,8^. In this study, we operationalized CR as memory reserve (i.e., memory-related CR), defined as attenuation of the CSF pTau181-memory association. CR has been studied using three broad approaches. First, the *proxies approach* estimates CR from activities thought to promote it, such as higher educational attainment, occupational complexity, or cognitively stimulating activities^9–12^. Second, the *residuals approach* estimates CR based on the amount of cognitive performance that remains unexplained after accounting for demographic factors and neuropathology burden^13,14^. Finally, the moderation approach, widely regarded as the most direct evidence of CR, tests whether a candidate CR factor attenuates the pathology-to-cognition association^7,15^.

CR is most informative at the prodromal stage of AD, where individuals with mild cognitive impairment (MCI) show substantial heterogeneity in disease progression. While many MCI patients experience rapid cognitive decline, a considerable proportion remain stable or even revert to normal cognition. It has been observed that higher CR is associated with preserved cognitive function, even after accounting for brain pathologies^16^.

Despite its recognized importance, the biological underpinnings of CR remain poorly understood. While a number of studies investigated CR using genetic and proteomic approaches^17–19^, few have examined its epigenetic basis. DNA methylation (DNAm) is the most extensively studied epigenetic mechanism, it is influenced by both genetic and environmental factors, including lifestyle factors such as smoking, diet, and exercise^20^, which have been shown to contribute to CR^21^.

In this study, we aimed to develop a blood DNAm signature of CR, by identifying CpGs that moderate the impact of AD pathology on memory. To this end, we analyzed 92 samples from the MCI subjects in Alzheimer’s Disease Neuroimaging Initiative (ADNI) study with matched blood-based DNA methylation, CSF pTau181 levels, and PHC_MEM harmonized memory scores measured at the same visit. Specifically, we modeled memory as a function of DNAm, pathology as quantified by CSF pTau181 levels, and their interaction, adjusting for age, sex, number of *APOE4* alleles, and major immune cell type proportions in the blood samples. We used CSF pTau181 as our index of tau pathology because it is sensitive to early disease-related changes, significantly associated with clinical cognitive symptoms, and correlates with greater baseline tau-PET signal, as well as faster longitudinal tau accumulation, especially among amyloid-positive subjects^22,23^.

To better understand the functional roles of CR-related DNAm changes, we performed integrative analyses leveraging multiple external resources, including eQTM (methylation and gene expression associations), mQTL (methylation and genetic variant associations), GWAS summary statistics, brain-blood correlations, and biological pathway databases. Furthermore, we evaluated the feasibility of DNAm as a biomarker for individualized prognosis in MCI, a stage when disease trajectories may still be modifiable.

## METHODS

### Study participants

We analyzed blood DNA methylation data from 92 amyloid-positive, self-identified non-Hispanic White participants who were diagnosed with MCI in the ADNI cohort. In addition to DNAm data, each of these subjects also had available data on CSF p-tau181 (PHC_pTau), memory composite scores (PHC_MEM), and amyloid positivity status, all generated by the Alzheimer’s Disease Sequencing Project Phenotype Harmonization Consortium (ADSP-PHC)^24^. Data was obtained from the ADNI portal (adni.loni.usc.edu).

### Cognitive measures and CSF biomarkers

PHC_MEM is a harmonized memory composite derived primarily from verbal learning and delayed-recall measures (e.g., WMS-R Logical Memory, Rey AVLT, and memory items from ADAS-Cog, and screening instruments), co-calibrated across ADNI, Adult Changes in Thought (ACT) and Religious Orders Study and Memory and Aging Project (ROS/MAP) cohorts using confirmatory factor analysis^24^.

The CSF AD biomarkers Aβ42, total tau, and pTau181 in ADNI were quantified using the Roche Elecsys platform^25^ and subsequently co-calibrated across ADSP-PHC cohorts by the ADSP-PHC consortium. Harmonization involved removing duplicate observations, excluding log10-scale outliers beyond the interquartile range (Q1−1.5 IQR or Q3+1.5 IQR), and standardizing the remaining values to z-scores to generate harmonized metrics comparable across ADNI, NACC, and Knight ADRC datasets^26^. Amyloid-positive (A+) status was then assigned using data-driven cut points from a two-component Gaussian mixture model applied to the post-QC z-scores of CSF Aβ42.

### DNA methylation data pre-processing

The analysis pipeline followed our established workflow^12^. CpG methylation was measured using the Illumina Infinium MethylationEPIC v1.0 Beadchip arrays. Supplementary Table 1 shows detailed probe and sample counts at each quality control (QC) step. For the QC of samples, we removed samples with low bisulfite-conversion efficiency (< 85%) or had discrepancies between the recorded sex and DNAm-predicted sex status. For the QC of probes, we removed probes lacking annotations, mapping to sex chromosomes, or whose IDs did not start with “cg”. Missing probe values, as well as probes with detection *P*-values > 0.01, were imputed using methyLImp2 R package^27^. We also removed CpGs that are cross-reactive^28^ or located close to single nucleotide polymorphism (SNPs).

Methylation beta values were normalized using β-mixture quantile normalization (BMIQ) as implemented in the watermelon R package^29^. Batch effects were corrected with Harman R package^30^. We removed outlier samples identified in principal component analysis (PCA), defined as those falling outside –3 standard deviations from the mean of PC1 and PC2. Restricting to samples from MCI subjects, our final DNAm dataset included 92 samples.

Next, using the quality controlled DNAm data, we estimated the immune cell-type proportions (B lymphocytes, natural killer cells, CD4+ T cells, CD8+ T cells, monocytes, neutrophils, and eosinophils) using the EpiDISH R package. Consistent with previous blood-based DNAm studies^31–33^, granulocyte proportions were computed as the sum of neutrophils and eosinophils proportions, as both cell types are classified as granular leukocytes.

### Statistical Models for identifying DNAm involved in memory reserve

We first regressed PHC_MEM scores on covariates (age, sex, number of *APOE4* alleles, estimated major immune cell type proportions) and used the residuals as covariate-adjusted memory scores. For each CpG, we then fit

Model 1: $$\text{memory}\;\text{score}\;\text{residual}=\alpha +\beta \text{pTau}181+\gamma \left(\text{DNAm}\times \text{pTau}181\right)+\tau \text{DNAm}+\varepsilon$$

where DNAm is DNA methylation beta value, pTau181 is CSF pTau181 level standardized to z-scores, and ε is residual error term.

A significant DNAm×pTau181 interaction (γ≠0) indicates that the association between pTau181 and memory depends on DNAm at that CpG. Assuming higher pTau181 is associated with worse memory (β<0), a positive γ indicates attenuation of the negative pTau181-memory association at higher DNAm, consistent with greater memory reserve. On the other hand, a negative γ indicates more negative pTau181-memory association at higher DNAm (i.e., less negative association at lower DNAm), where greater memory reserve corresponds to lower DNAm. The pTau181 slope at a given DNAm level is estimated by *β* + γDNAm.

### Inflation assessment and correction

We estimated genomic inflation factor (lambda value) using the *bacon* method, which was specifically designed for EWAS^34^. The estimated inflation factor was 1.06 and the estimated bias was −0.058. After genomic correction using the bacon method, the estimated inflation factor and bias were 1 and 9.90×10^−7^, respectively. The bacon method was then used to compute bacon-corrected effect sizes, standard errors, and *P*-values for the DNAm × pTau181 interaction. To exclude CpGs with minimal variability, we required an interquartile range (IQR) greater than 0.02 in beta values across all samples. We considered CpGs with *P*-values less than 1 × 10^−5^ to have suggestive significance. To account for multiple comparisons, we computed False Discovery Rate (FDR).

### Differentially methylated regions analysis

For region-based analysis, we used the comb-p method^35^, which scans genome-wide CpG locations and *P*-values to identify regions enriched with clusters of low *P*-values. We used *P*-values of the DNAm × pTau181 interaction effect as input for comb-p and parameter settings with --seed 0.05 and --dist 750 (a *P*-value of 0.05 is required to start a region and extend the region if another *P*-value was within 750 base pairs). These parameters were shown to have optimal statistical properties in our previous comprehensive assessment of the comb-p software^36^. As comb-p uses the Sidak method to account for multiple comparisons, we selected differentially methylated regions (DMRs) with Sidak *P*-values less than 0.05. To further reduce false positives, we also required all the CpGs within the DMR to have a consistent direction of change.

### Functional annotation and pathway analysis

Significant individual CpG methylation signals and DMRs were annotated using gene annotations from Illumina and the Genomic Regions Enrichment of Annotations Tool (GREAT) software^37^, which associates genomic regions with target genes.

To identify biological pathways enriched for CR-related DNAm, we used the methylRRA function from the methylGSA R package^38^, which analyzes single CpG *P*-values as input. Briefly, methylGSA computes a gene-wise ρ value by aggregating *P*-values from multiple CpGs mapped to each gene, adjusts for the different numbers of CpGs per gene using Bonferroni correction, and then performs Gene Set Enrichment Analysis^39^ in pre-ranked mode to identify pathways enriched with significant CpGs. We analyzed GO terms from the KEGG and REACTOME databases, restricting analyses to pathways containing between 5 and 200 genes. To avoid gene sets where the enrichment signal is driven by only one or two genes, we additionally required that significant gene sets include at least three genes in the “core enrichment” subset.

### Integrative analyses with gene expression, genetic variants, and brain-to-blood correlations

To evaluate the effect of the significant DNAm on the expression of nearby genes in blood samples, we overlapped our CR-related DNAm, including both significant individual CpGs and those located within DMRs, with eQTm analysis results in Supplementary Tables 2 and 3 of Yao et al. (2021)^40^.

To assess the correlation of DNAm levels between blood and brain samples at CR-related CpGs, we used the London dataset, which includes 69 matched samples from blood and brain prefrontal cortex tissues^41^. Brain-blood correlations were assessed using two approaches: (1) unadjusted correlations based on methylation beta values and (2) adjusted correlations using residuals from regression models accounting for covariates. In unadjusted analysis, we calculated Spearman rank correlations between DNA methylation beta values measured in brain and blood samples. For adjusted analysis, we accounted for potential confounders by removing the effects of covariates. Specifically, we removed effects from estimated neuron proportions in brain samples (or estimated immune cell-type proportions in blood samples), batch, age at death (for brain samples) or age at blood draw (for blood samples), and sex, by fitting linear models separately for brain and blood samples and extracting residuals. Spearman correlations were then calculated using these residual values to assess the relationship between brain and blood methylation levels independent of confounders.

For correlation and overlap with genetic loci, we searched blood mQTLs using the GoDMC database (http://mqtldb.godmc.org.uk/downloads).^42^ Using the same criteria from the original GoDMC study,^42^ we considered a cis *P*-value smaller than 10^−8^ and a trans *P*-value smaller than 10^−14^ as significant. The genome-wide summary statistics for genetic variants associated with dementia described in Bellenguez et al. (2022)^43^ were obtained from the European Bioinformatics Institute GWAS Catalog (https://www.ebi.ac.uk/gwas/) under accession no. GCST90027158.

### Computation of Methylation Reserve Scores (MRS) and assessment of its utility in predicting future memory decline

The MRS was computed based on the 6 significant individual CpGs and 88 CpGs located within DMRs. In Model 1 described above, for each subject and at each CpG, pTau effect on memory at a given DNAm level is estimated as *β*_pTau_ + γ_pTau× DNAm_*M*, where *β*_pTau_ is the estimated main effect of pTau, γpTau× DNAm is the interaction effect, and *M* is methylation beta value. We then averaged the CpG-specific values across the 94 CpGs to obtain a subject-specific raw score (MRS_raw_), so that lower values indicate greater pTau-associated memory decline. It was then rescaled as a z-score using the scale () function.

To evaluate if the MRS associates with future memory decline, we fitted the mixed-effects model below (Model 2), using the lmerTest R package, to the 88 ADNI subjects with longitudinal follow up information.


Model 2: $$\text{PHC}\_\text{MEM}\sim \text{baseline}\_\text{age}+\text{PHC}\_\text{Sex}+\text{APOE}4+\text{PHC}\_\text{Education}+\text{MRS}+\;\text{time}+\text{baseline}\_\text{pTau}+\text{MRS}*\text{time}+\text{baseline}\_\text{pTau}*\text{time}+\;\text{baseline}\_\text{pTau}*\text{MRS}+\left(1|\text{RID}\right)$$

## RESULTS

### Study cohort

To identify DNAm signatures for memory reserve, we analyzed a cross-sectional dataset that included CSF pTau181 biomarker, memory assessments, and blood DNAm measured at the same visit. Our analysis included 92 amyloid positive individuals diagnosed with MCI from the ADNI cohort. Participants had a mean age of 74.67 ± 7.51 years; 44.57% (41 subjects) were women, and the mean duration of education was 16.21 ± 2.78 years. Approximately half (52%, 48 subjects) carried at least one *APOE* ε4 allele, and 43% (40 subjects) reported a history of smoking. Baseline CSF pTau181 biomarker concentration was 46.46 ± 27.32 pg/mL (Table 1).

### The CSF pTau181-memory association are dependent on DNA methylation at individual CpGs and DMRs

**Figure 1:**
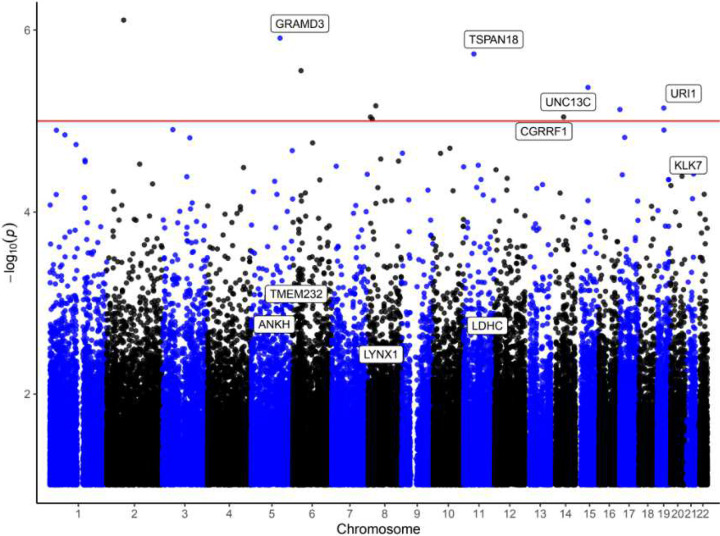
Manhattan plot of DNAm × pTau181 interaction results (Model 1) in MCI subjects from ADNI study. The x-axis shows chromosomes 1–22 and the y-axis shows -log_10_ (*P*-value). The horizontal red line marks the suggestive significance threshold (*P*-value < 1× 10^−5^).

After regressing PHC_MEM on covariate (age, sex, *APOE ε4* allele count, and immune cell type compositions), we used the residuals as covariate-adjusted memory scores and fit Model 1 at each CpG, testing DNAm×pTau181 interaction (with corresponding main effects of DNAm and pTau181). After correcting for genomic inflation, we identified 11 CpGs with a DNAm×pTau181 interaction effect meeting the suggestive significance threshold (*P* < 1×10^−5^); none reached the 5% FDR significance. After excluding low-variability CpGs (IQR < 0.02 in beta values across all samples), 6 CpGs remained. Among them, 1 CpG (cg03281038) was located in the promoter region of *UNC13C* gene. Four CpGs were located in the gene bodies of *GRAMD3, TSPAN18, CGRRF1*, and *URI1* genes, and 1 CpG was located in an intergenic region (Table 2, Figure 1, Supplementary Figure 1).

Across the 6 CpGs with suggestive DNAm × pTau181 interaction effects, DNAm moderated the association between pTau and memory, with the strength of the association varying by CpG-specific methylation level. Four CpGs showed positive interaction estimates, such that higher DNAm was associated with a weaker (less negative) pTau-memory relationship. In contrast, for two CpGs (cg01602139 and cg11145222), the interaction estimates were negative, indicating that lower DNAm was associated with a weaker pTau181- memroy relationship (i.e., greater memory reserve).

**Figure 2:**
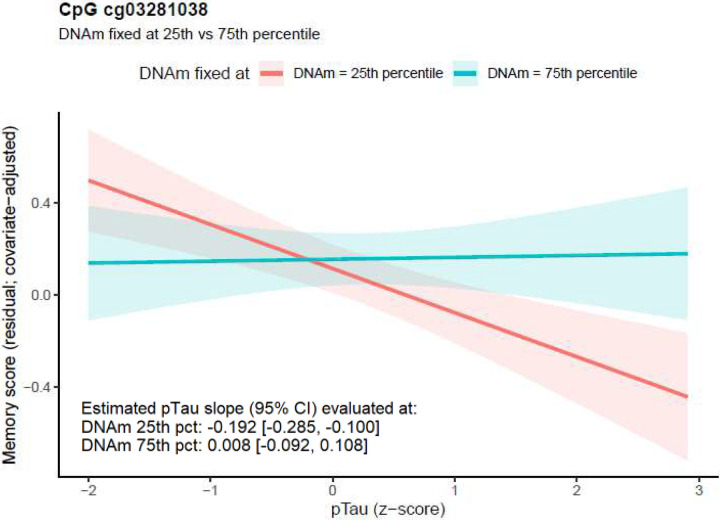
Estimated pTau-memory association (with 95% CI) from Model 1, evaluated with DNAm at cg03281038 (in UNC13C promoter) fixed to the 25th versus 75th percentile. At DNAm fixed to the 25th percentile, higher pTau was associated with poorer memory (β = −0.192, 95% CI: −0.285 to −0.100). In contrast, at DNAm fixed to the 75th percentile, the pTau-memory slope was near zero (β = 0.008, 95% CI: −0.092 to 0.108), indicating attenuation of the pTau-memory association and supporting a DNAm×pTau moderation effect.

For example, at cg03281038 in the *UNC13C* promoter region, the pTau memory association varied by DNAm level. When DNAm was fixed at the 25^th^ percentile, higher pTau was significantly associated with poorer memory performance (β = −0.192, 95% CI −0.285 to −0.100). In contrast, when DNAm was fixed at the 75^th^ percentile, the pTau-memory slope was near 0 (β = 0.008, 95% CI: −0.092 to 0.108), indicating attenuation of pTau-memory association and supporting a moderation effect (Figure 2). *UNC13C* encodes a presynaptic protein that primes synaptic vesicles and is essential for neurotransmitter release, a process supporting synaptic function and neural communication that has been repeatedly implicated in cognitive resilience^44^.

**Figure 3:**
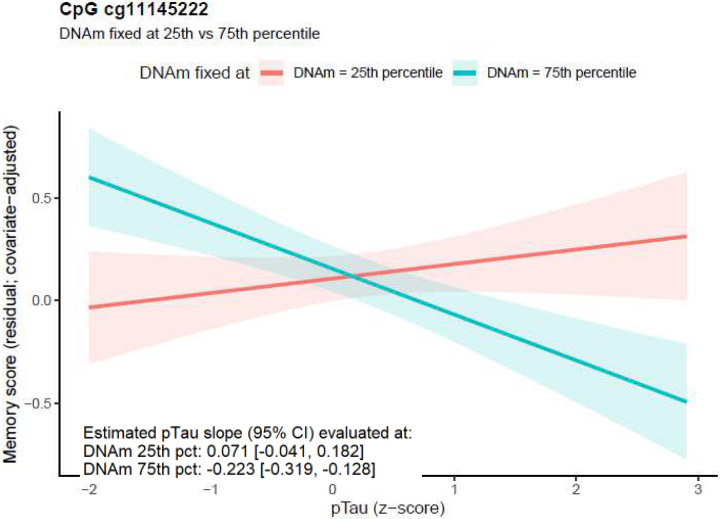
Estimated pTau- memory association from Model 1 for CpG cg11145222 located in the *TSPAN18* gene, evaluated with DNAm fixed to the 25th versus 75th percentile. Lines show the fitted pTau slope at each fixed DNAm level (shaded bands: 95% CI). At DNAm fixed to the 25th percentile, the estimated pTau slope was 0.071 (95% CI: −0.041 to 0.182), while at DNAm fixed to the 75th percentile, the slope was −0.223 (95% CI: −0.319 to −0.128), indicating a stronger negative pTau-memory association at higher DNAm and attenuation at lower DNAm, consistent with a DNAm×pTau moderation effect.

Figure 3 shows a CpG with a negative DNAm × pTau181 interaction estimate. At cg11145222 located in *TSPAN18*, when DNAm was fixed at the 75^th^ percentile, higher pTau181 was associated with poorer memory (β = −0.223, 95% CI: −0.319 to −0.128). In contrast, when DNAm was fixed at the 25^th^ percentile, the pTau-memory association was not significant (β = 0.071, 95% CI: −0.041 to 0.182), indicating attenuation at lower DNAm. Biologically, *TSPAN18* has been implicated in endothelial calcium signaling and thrombo-inflammatory responses, processes contributing to vascular and blood-brain barrier (BBB) integrity^45^. Impairment of vascular and BBB integrity has been repeatedly associated with cognitive decline, aging, and AD pathology^46^.

Interestingly, we found that none of these CpGs showing a significant DNAm × pTau interaction had marginal associations with CSF pTau181 or memory that survived Bonferroni correction; the only nominal signal was for cg26869675 with memory (*P*-value = 0.038), which did not remain significant after multiple-testing correction (Supplementary Figure 2).

Using *P*-values for individual CpGs as input, comb-p software^35^ identified 11 DMRs, which achieved both a nominal *P*-value < 1×10^−5^ and a Sidak multiple comparison-adjusted *P*-value < 0.05. We also required all the CpGs within each DMR have a consistent direction of change in estimated effect sizes in the individual CpG analysis (Table 3). The number of CpGs in these DMRs ranged from 3 to 17. Among these DMRs, the majority (7 DMRs, 64%) showed hypermethylation associated with greater memory reserve. A total of 5 DMRs (45%) are in promoter regions of the genes *KLK7, TMEM232, ANKH, LYNX1*, and *LDHC*. As with the significant individual CpGs, we found that all DMRs, except one located in gene body of *JAKMIP3*, showed no significant association with either CSF pTau181 or memory (Supplementary Figure 3).

Because 43% of participants reported a history of smoking, we refitted the models with additional adjustment for smoking status. Effect estimates and *P*-values were very similar, and all CR-related CpGs and DMRs remained highly significant (Supplementary Table 2), indicating that these associations are unlikely to be explained by smoking history.

### Pathway analysis revealed memory reserve-related DNA methylation are enriched in biological pathways involved in metabolic health and inflammation

To further understand the biological processes underlying cognitive reserve, we performed pathway analysis using the methylGSA software which used *P*-values from all the CpGs as input.^38^ We applied a 25% false discovery rate (FDR) threshold, an alternative to the conventional 5% FDR that has been suggested for GSEA.^47^ At this threshold, we identified 2 KEGG and 20 Reactome pathways significantly enriched for memory reserve-related DNA methylation (Supplementary Table 3). The most significant pathway was the KEGG *adipocytokine signaling pathway* (*P*-value = 2.6 × 10^−4^, FDR = 0.056), which represents the network of hormones and cytokines released by adipose tissue, most prominently leptin and adiponectin. Leptin enhances synaptic plasticity in the hippocampus, promotes β-amyloid clearance, and improves memory performance in animal models of aging and AD.^48^ In the Framingham Heart Study, higher plasma leptin levels were associated with a lower risk of incident dementia and with favorable brain health indices.^49^ Adiponectin, on the other hand, exerts insulin-sensitizing, anti-inflammatory, and antioxidant effects in peripheral tissues, functions that may also provide protection against neurodegenerative processes such as AD.^50^

Similarly, several significant Reactome pathways were also related to adipose and metabolic health. These included *Regulation of lipid metabolism by PPARα* (a lipid-sensing signaling cascade that enhances fatty acid oxidation and energy metabolism), *Triglyceride catabolism* (breakdown of stored fat to provide energy), *Adipogenesis* (formation of mature fat cells capable of safely storing and releasing lipids), *Transcriptional regulation of white adipocyte differentiation* (activation of gene programs that produce insulin-sensitive white adipocytes), and *Epigenetic regulation of adipogenesis genes by MLL3 and MLL4 complexes* (histone-modifying complexes that epigenetically control metabolic and inflammatory gene expression).

Taken together, these results highlighted the importance of maintaining metabolic balance, preventing lipid accumulation in the brain and peripheral tissues where it can trigger inflammation, preserving insulin sensitivity, and reducing systemic metabolic stress that may worsen neuropathology.^51^ Balanced adipose and lipid signaling pathways support healthy lipid handling and anti-inflammatory responses, which in turn may help preserve brain function and promote cognitive resilience in the presence of neurodegenerative pathology.

### Correlation of significant DNAm with expression of nearby genes in blood samples

To evaluate the functional relevance of our significant DMRs and CpGs involved in memory reserve, we compared them with established DNAm to gene-expression associations (i.e., eQTMs) computed from matched blood DNAm and gene-expression data in over 4000 participants from the Framingham Heart Study^40^. Among the 6 significant individual CpGs and 88 CpGs within the 11 DMRs, 3 CpGs in the promoter region of *ANKH* and 6 CpGs in the promoter region of *LDHC*, all of which were located in DMRs, showed significant *cis* (within 500 kb) associations with gene expression levels (Supplementary Table 4).

Notably, the *ANKH* gene encodes a transmembrane protein that regulates extracellular mineralization by controlling the export of key metabolites such as ATP and citrate. A recent genome-wide association study (GWAS) identified a protective allele (rs112403360) in *ANKH* that is strongly associated with cognitive resilience and a reduced risk of AD among cognitively healthy centenarians^52^. Impaired *ANKH* function can lead to pathological calcification, including arterial and vascular mineralization. The protective allele, as well as the hypomethylation we observed in the promoter of this gene, may help preserve vascular integrity and reduce neuropathological burden, thereby supporting sustained cognitive function.

At the other eQTM locus, the *LDHC* gene is mainly expressed in testis. However, its paralog *LDHA*, which shares high sequence similarity and is located in proximity (< 7 kb), is ubiquitously expressed in somatic tissues, including blood and brain. Because both DNAm and gene expression in the eQTM resource were measured using expression arrays, which are susceptible to cross-hybridization between paralogous genes, the observed *LDHC* eQTM likely reflects transcriptional activity at the broader *LDHC/LDHA* locus, and may be driven predominantly by *LDHA* expression. *LDHA* encodes lactate dehydrogenase A, which produces lactate, a key neuronal energy substrate essential for long-term memory formation^53^. Although *LDHA*-associated lactate production is required for normal neuronal plasticity, studies in animal models of aging suggest that sustained alterations in neuronal *LDH/LDHA* activity and a shift toward higher brain lactate are associated with shortened lifespan, neurodegeneration, and age-related memory impairment, suggesting that tight regulation of this locus is important for cognitive resilience^54^.

### Methylation quantitative trait loci and intersection with genetic risk loci in dementia

To identify methylation quantitative trait loci (mQTLs) for the significant DMRs and CpGs, we next performed look-up analyses using the GoDMC database^42^ for mQTLs. Among the 6 significant CpGs and 88 CpGs located in the DMRs, 64 and 21 CpGs had mQTLs in *cis* and in *trans*, respectively (Supplementary Table 5).

Next, to evaluate if the significant mQTLs overlapped with genetic risk loci implicated in AD, we compared the mQTLs with genetic variants nominated by a recent Alzheimer’s Disease and Related Dementias (ADRD) GWAS meta-analysis.^43^ We found 237 mQTLs associated with DNAm at 16 significant CpGs (all located within DMRs) overlapped with genetic variants reaching suggestive significance (*P*-value < 1 × 10^−5^). Among them, 65 mQTLs associated with DNAm at the same 16 significant CpGs, are in high LD with genome-wide significant loci rs6605556 (*P*-value = 7.1 × 10^−20^) within the HLA region on chr6:32,395,036–32,636,434, which included *HLA-DRA, HLA-DRB5, HLA-DRB1, HLA-DQA1, and HLA-DQB1* genes (Supplementary Table 6).

### Correlation of DNAm levels in blood and brain samples at memory reserve related CpGs

We next evaluated cross-tissue concordance of DNAm levels at significant CpGs using the London dataset, which includes matched pre-mortem blood and post-mortem prefrontal cortex DNAm profiles from 69 subjects^41^. At 5% FDR, among the 6 significant individual CpGs and 88 CpGs mapped within the identified DMRs, 46 CpGs (48.9%) showed significant brain-blood correlations in both unadjusted and adjusted analyses that accounted for covariate variables (Supplementary Table 7). These CpGs are located in promoter regions of the *LDHC, KLK7, ANKH, TMEM232, LYNX1* genes and intergenic regions. All CpGs showed positive brain-blood methylation correlations.

### Methylation-based memory reserve biomarker is significantly associated with future memory decline in MCI subjects

To evaluate feasibility of leveraging cognitive reserved-related DNAm to predict future memory decline, we developed a methylation reserve score (MRS) from CpGs with significant DNAm × pTau interactions, including 6 individual CpGs and 88 CpGs located within DMRs. For each subject and at each CpG, the pTau effect on memory is computed as *β*_pTau_ + *γ*_pTau× DNAm_*M*, where *β*_pTau_ is the estimated main effect of pTau, and *γ*_pTau× DNAm_ is the estimated interaction effect, and *M* is methylation beta values in Model 1 (Methods). For each subject, the MRS was then computed by averaging these CpG-specific values across all 94 selected CpGs. Note that the MRS reflects the DNAm-dependent effect of pTau for each subject, and lower MRS values indicate greater pTau-associated memory decline (i.e., a more negative pTau effect on memory), while higher values indicate attenuation of pTau’s impact (i.e., less negative pTau effect on memory, thus more memory reserve).

Among 92 MCI subjects with DNA methylation, PHC_MEM, and CSF pTau measured at the same visit (defined as baseline visit), 88 had at least one follow-up memory assessment. We restricted the longitudinal dataset to follow-up visits occurring after the baseline visit and fitted linear mixed-effects models to assess the association between baseline MRS and longitudinal memory (PHC_MEM). Random intercept effects for each subject were included to account for within-subject correlations among repeated measures. We adjusted for baseline pTau, age, sex, education, *APOE4*, and included interaction effects MRS × time, pTau × time, and pTau × MRS. The interpretations of the mixed effects model coefficients are presented in Table 4. Note that baseline age, pTau and MRS were standardized (z-scored), so that their mean baseline values are 0.

Our results showed that memory declined significantly over time (β_time_ = −0.050 per year, *P*-value = 1.8 × 10^−9^) (Table 5). At baseline (time = 0, average MRS and pTau), older age and *APOE4* carrier status are significantly associated with lower memory (β_age_ = −0.308, *P*-value = 6.1 10^−6^; β_APOE4_ = −0.480, *P*-value = 2.2×10^−5^), while sex, education and the main effect of MRS were not significant. Higher baseline pTau is associated with lower baseline memory (β = −0.138, *P*-value = 0.025); it also predicted faster decline (β_time × pTau_ = −0.026 per year per 1 unit increase in pTau, *P*-value = 3.0 × 10^−4^). At baseline, MRS attenuates the harmful effect of pTau on memory (β_MRS × pTau_ = 0.217, *P*-value = 2.4 × 10^−4^). Importantly, higher MRS at baseline is significantly associated with slower decline (β_MRS × time_ = 0.034, *P*-value = 9.5 × 10^−5^). Taken together, these results indicate that higher MRS corresponds to greater memory reserve, reflected by slower memory decline and reduced pTau-related memory impairment at baseline.

**Figure 4:**
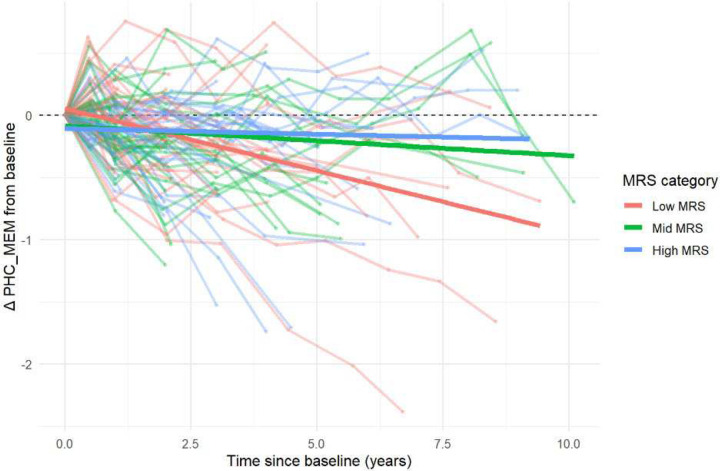
Longitudinal change in memory by methylation reserve score (MRS) tertile. Trajectories show change in harmonized memory (PHC_MEM) scores from baseline among amyloid positive ADNI subjects with mild cognitive impairment (MCI), grouped by tertiles of baseline MRS. Thin lines represent individual subjects, and thick lines indicate fitted linear regression slopes for each MRS group. Participants with higher MRS showed slower memory decline over time, consistent with greater cognitive reserve.

Figure 4 shows changes from baseline in harmonized memory scores for the 88 MCI subjects, grouped by tertiles of their baseline MRS. Notably, subjects with higher MRS showed slower memory decline over time. To formally test group differences, we refit the mixed model described above, except by replacing MRS scores with MRS tertile indicators. Our results showed that compared to the high-MRS group, the low-MRS group declined significantly faster (β_low MRS × time_ = −0.053, *P*-value = 0.008) and showed a stronger harmful baseline pTau-to-memory association (β_low MRS×pTau_ = −0.520, *P*-value = 4.1×10^−4^); the mid- vs. high-MRS comparison was not significant (Supplementary Table 8). Overall, higher baseline MRS is associated with more attenuation in tau-related effects on memory at baseline, as well as slower longitudinal memory decline, consistent with greater memory reserve.

## DISCUSSION

Consistent with the consensus that conceptualizes CR as a moderator of pathology-cognition relationships^7,8^, we operationalized CR specifically as memory reserve and performed comprehensive interaction-based analyses to identify a blood DNAm signature of CR that modifies the association between CSF pTau181 and memory in amyloid positive ADNI participants with MCI. Across the epigenome, we identified 11 CpGs showing a DNAm×pTau181 interaction at a suggestive significance threshold (*P*-value < 1×10^−5^), and 11 DMRs significant after multiple comparison correction. After excluding low-variability CpGs (IQR < 0.02), 6 CpGs remained.

Notably, the direction of memory reserve, defined as attenuation of the pTau181-memory association, was locus-specific. At some sites, higher methylation was associated with weaker pTau181-memory association, while at others, lower methylation was protective. This heterogeneity is consistent with context-dependent DNAm regulation, in which hyper- and hypomethylation may have beneficial or adverse effects depending on genomic and regulatory context. Moreover, a composite methylation reserve score derived from these loci was significantly associated with slower longitudinal memory decline in MCI, independent of baseline pTau181, age, sex, *APOE* ε4, and education. Together, these findings support blood DNAm as a promising biological index of CR with potential clinical utility in the prodromal phase of AD.

A key feature of these CR-related loci is that, despite showing robust DNAm×pTau181 interaction effects, nearly all lacked strong marginal associations with pTau or memory (Supplementary Figures 2–3). Therefore, these DNAm do not simply reflect tau burden, nor do they serve primarily as biomarkers of current memory performance. Instead, they mark additional variations that determine how cognition varies about a given level of tau pathology. The lack of strong marginal effects further argues against a mediating role of DNAm in the pTau-memory relationship, but instead supports a moderating role, in which DNAm at these loci, or the biological mechanisms they mark (e.g., transcription factor binding, chromatin accessibility, histone modifications), influences the impact of tau pathology on memory. Future mechanistic studies are needed to determine how DNAm, or the regulatory elements it marks, modulates CR-related biological processes.

Several of the most significant loci point to biologically plausible mechanisms for cognitive reserve. The most significant DMR is located in the promoter of the *KLK7* gene, which encodes an astrocyte-derived protease, an enzyme helps break down and clear deposited amyloid beta in the brain. Loss of *KLK7* activity has been shown to exacerbate amyloid pathology in mouse models of AD, suggesting it plays a protective role in maintaining brain health^55^. Consistent with these findings, we observed hypomethylation in in the promoter of the *KLK7* gene associated with greater memory reserve.

The second most significant promoter-associated DMR is located in the *TMEM232* gene, which encodes a transmembrane protein that promotes inflammatory responses to atopic dermatitis, and has been implicated by genetic studies in allergic and atopic diseases characterized by immune dysregulation^56^. Importantly, recent DNAm studies in blood have also identified DMRs at *TMEM232* associated with MCI, AD, as well as CSF AD biomarkers^57–59^. Consistent with these findings, the hypermethylation we observed at *TMEM232* associated with greater CR may suggest that reducing *TMEM232*-mediated inflammatory signaling could contribute to a peripheral immune environment that mitigates the impact of AD pathology.

Finally, the DMR annotated to the *LYNX1-SLURP2* read-through transcript, which spans the adjacent *LYNX1* and *SLURP2* genes, is involved in regulation of nicotinic acetylcholine receptor (nAChR) activity within the cholinergic system. In animal models, loss of *LYNX1* leads to age-related neurodegeneration, highlighting its critical role in maintaining neuronal health during aging^60^. Moreover, treatment with a water-soluble *LYNX1* analogue was shown to prevent amyloid beta-induced blockade of memory-related synaptic plasticity, indicating *LYNX1* may be a potential therapeutic target for improving cognitive deficits in neurodegenerative diseases^61^. These findings are consistent with our observed hypomethylation at this locus associated with greater CR.

To help prioritize the most biologically relevant loci involved in CR, we next performed integrative analyses incorporating several external resources, to better understand the functional roles of the CR-related DNAm changes. These analyses nominated the *ANKH* and *LDHA/LDHC* loci, where DNAm was associated with nearby gene expression (Supplementary Table 4). Recent genetic studies have implicated *ANKH* in cognitive resilience and reduced AD risk, and impaired *ANKH* function is associated with pathological vascular mineralization^52^. The observed hypomethylation at *ANKH* promoter associated with greater CR may therefore reflect epigenetic upregulation of a protective vascular pathway, which helps to limit microvascular damage and neuropathological burden.

At the *LDHC* locus, the proximity and high sequence similarity between *LDHC* and its paralog *LDHA* suggest that the observed eQTM likely reflects transcriptional activity at the broader *LDHC/LDHA* locus. *LDHA* is a key enzyme in lactate production, and lactate serves as an important neuronal energy substrate that is critical for memory formation but may become maladaptive when chronically elevated in aging and neurodegeneration^53^. The association between CR-related methylation and *LDH/LDHA* activity is therefore consistent with the idea that tight regulation of neuronal energy metabolism and lactate homeostasis is important for preserving cognitive function in the presence of tau pathology. Intriguingly, our blood-to-brain correlation analysis also showed several CpGs in the *LDHC/LDHA* DMR showed strong positive correlations between blood and brain methylation, suggesting this locus may be a promising candidate for a blood-based biomarker of memory reserve.

Our pathway analysis of genes mapped to CR-related CpGs extended these gene-level observations and highlighted a strong enrichment of metabolic pathways, particularly those involving adipocytokine signaling, lipid handling, and adipose tissue function (Supplementary Table 3). The identification of the KEGG adipocytokine signaling pathway, along with Reactome pathways related to PPARα activation, triglyceride catabolism, adipogenesis, and epigenetic regulation of adipocyte differentiation, suggests that systemic metabolic health and lipid homeostasis are key biological processes involved in CR. Our findings support a model in which CR-related DNAm captures, at least in part, long-term exposure to metabolic states that maintain insulin sensitivity, prevent lipid accumulation, and reduce chronic inflammation. In turn, these favorable metabolic profiles may help preserve neural networks and reduce impact of accumulating tau pathology on memory.

A major strength of this study is the construction and evaluation of an MRS that aggregates information across multiple CR-related CpGs and DMRs into a single, interpretable measure. By definition, this score quantifies the DNAm-dependent effect of pTau on memory, such that higher values represent greater attenuation of tau’s harmful impact. In longitudinal mixed-effects models, a higher MRS at baseline was significantly associated with slower subsequent decline in harmonized memory scores among MCI participants, even after adjusting for age, sex, education, APOE ε4, and baseline pTau. Moreover, individuals in the lowest MRS tertile experienced both a stronger pTau-memory association at baseline and a faster rate of memory decline compared with those in the highest tertile (Supplementary Table 8). These findings demonstrate that CR-related DNAm patterns are not merely cross-sectional correlates but provide prognostic information about future clinical trajectories in prodromal AD. If replicated in larger and more diverse cohorts, MRS or similar DNAm-based metrics could be used to stratify individuals at baseline, and refine prognosis beyond traditional biomarkers, and identify those most likely to experience rapid decline.

Our results are consistent with prior work associating CR with modifiable lifestyle and cardiometabolic factors^21 ,62^. A number of the pathways enriched with CR-related DNAm in our study, including those involved in adipocytokine signaling, lipid metabolism, and vascular function, have previously been associated with smoking, obesity, physical inactivity, and other lifestyle factors in large epigenome-wide association studies^63,64^. This suggests that DNAm may serve as a biomarker capturing, at least in part, the cumulative impact of life-course exposures that build or erode reserve. Moreover, emerging evidence shows that lifestyle-based interventions can modify DNAm at cardiometabolic loci^65,66^, further supports the idea that CR is not a fixed trait determined only by early-life education or genetics, but a dynamic, biologically embedded property shaped by experiences and behaviors across the lifespan^7^. In this context, DNAm represents a dynamic and potentially reversible biomarker that not only reflects prior exposures but may also track response to lifestyle interventions.

Several limitations should be considered when interpreting these findings. First, because we operationalized reserve using PHC_MEM (a harmonized memory composite), our findings pertain to memory reserve and may not generalize to reserve in other cognitive domains (e.g., executive function, language, global cognition). Second, our analysis was conducted in a relatively small sample of ADNI MCI participants with concurrent CSF, DNAm, and memory data, which limits statistical power to detect small effects and may contribute to the fact that no CpG passed a stringent FDR threshold at the single-site level. We therefore focused on loci meeting a suggestive *P*-value threshold and on DMRs, which leverage spatial correlation across neighboring CpGs; nonetheless, replication in independent cohorts is essential. Third, the ADNI cohort is highly educated and predominantly of European ancestry, and our results may not generalize to more diverse populations or to individuals at different stages of the AD disease course. Fourth, although we adjusted for major immune cell proportions and key demographic (age, sex) and genetic covariates, residual confounding by unmeasured lifestyle factors or additional neuropathologies cannot be excluded. To help mitigate inflation and bias related to unmeasured confounding, we used the bacon method to empirically calibrate test statistics^34^. Finally, the use of CSF pTau181 as the pathology term in the interaction model focuses the analysis on tau-related processes that are closely associated with amyloid-β deposition, and may not capture reserve mechanisms that primarily operate through vascular lesions, TDP-43, or other non-AD pathologies.

In conclusion, this study provides convergent evidence that blood DNAm captures biologically meaningful variation in memory reserve among individuals with MCI. By focusing on DNAm×pTau interactions, we identified CpGs and DMRs that moderate the impact of CSF pTau181 on memory, mapped these loci to vascular, synaptic, metabolic, and immune pathways, and integrated them into an MRS that predicts future memory decline. These findings highlight the promise of blood DNAm as a non-invasive biomarker for resilience to AD pathology and suggest that interventions targeting vascular and metabolic health, along with traditional risk-reduction strategies, may enhance reserve and delay clinical progression in at-risk older adults.

## Supplementary Material

Supplementary Files

This is a list of supplementary files associated with this preprint. Click to download.

- ALLSUPPTABLES12152025.xlsx
- ALLSUPPFIGURES12152025.pdf

## Figures and Tables

**Table 1:** Demographic and clinical characteristics of the prodromal (Aβ+ MCI) subjects in ADNI

Characteristics	MCI subjects in ADNI (n = 92)
Age, av. (sd)	74.67 (7.51)
Sex, n (%)	
Female	41 (44.57)
Male	51 (55.43)
APOE ε4, n (%)	48 (52.17)
Mean education year, av. (sd)	16.21 (2.78)
Smoking history, n (%)	
Yes	40 (43.48)
CSF pTau181 in pg/mL, av. (sd)	46.46 (27.32)

**Table 2:** Among amyloid-positive mild cognitive impairment (MCI) participants, 6 CpG sites showed suggestive evidence of moderating the effect of pTau on memory (DNAm × pTau interaction; *P*-value < 1×10^−5^) after excluding low-variability CpGs (IQR < 0.02 in beta values across all samples). The harmonized memory scores (PHC_MEM) were first regressed on age, sex, APOE ε4 allele count, and estimated immune cell-type proportions, the residuals were then used as the outcome. The linear model included memory residuals (outcome) and harmonized CSF pTau181, DNAm beta values, and their interaction. For each CpG, annotations include chromosome and position; nearby genes from GREAT (GREAT_annotation) and Illumina (UCSC_RefGene_Name); and the associated genomic feature (UCSC_RefGene_Group). Model results include the estimated effect size (estimate; positive values indicate that higher methylation is associated with greater memory reserve), its standard error (stdErr), and *P*-value (pValue). In GREAT annotations, numbers in parentheses denote distance to the nearest TSS.

	Annotations					Effect of DNAm × pTau interaction		
CpG	Chr	Position	GREAT_annotation	UCSC_RefGene_Name	UCSC_RefGene_Group	Estimate	StdErr	pValue
cg01602139	chr6	36,672,171	CDKN1A (-6538);SRSF3 (+77805)			−0.020	0.004	2.80E-06
cg03281038	chr15	54,013,008	UNC13C (+106)	UNC13C	1stExon	0.064	0.014	4.28E-06
cg08579041	chr5	126,364,531	GRAMD3 (+4401);ALDH7A1 (+230886)	GRAMD3	Body	0.072	0.015	1.23E-06
cg11145222	chr11	44,882,533	TP53I11 (+67626);TSPAN18 (+118110)	TSPAN18	Body	−0.032	0.007	1.83E-06
cg24481207	chr14	54,537,562	SAMD4A (-30354);CGRRF1 (+27736)	CGRRF1	Body	0.045	0.010	9.02E-06
cg26869675	chr19	30,000,747	ZNF536 (-371664);URI1 (+58498)	URI1	Body	0.051	0.011	7.19E-06

**Table 3:** Significant memory reserve-related DMRs at 5% Sidak adjusted *P*-value (z_sidak_p). For each DMR, annotations include location of the DMR, nearby genes based on GREAT (GREAT_annotation) and Illumina gene annotations (UCSC_RefGene_Name). Direction indicates sign of DNAm × pTau181 interaction at a particular CpG located within the DMR, that is, “+” sign corresponds to higher methylation at the CpG is associated with greater memory reserve, “-” sign corresponds to lower methylation at the CpG is associated with greater memory reserve. Highlighted in red text are promoter regions associated with DMRs.

DMR	nProbes	pValue	Sidak-*P*	direction	GREAT_annotation	UCSC_RefGene_Name
chr19:50983852-50984249	12	7.92E-12	1.55E-08	------------	KLK7 (-235)	KLK7
chr6:32095996-32096480	17	2.68E-11	4.30E-08	+++++++++++++++++	TNXB (-50110);ATF6B (+32002)	TNXB
chr5:110726641-110727135	13	6.48E-11	1.02E-07	+++++++++++++	TMEM232 (-223)	TMEM232
chr4:183987356-183987864	9	2.42E-10	3.70E-07	+++++++++	STOX2 (+82254);ENPP6 (+230351)	STOX2
chr6:7468547-7468818	5	4.13E-08	1.18E-04	+++++	CAGE1 (-78939);DSP (-72893)	
chr13:75870661-75870928	7	5.38E-08	1.57E-04	+++++++	LMO7 (+234136)	LMO7DN
chr8:142778252-142778573	8	3.64E-07	8.81E-04	--------	LYNX1-SLURP2 (-188)	LYNX1
chr10:132142481-132142678	3	1.67E-06	6.57E-03	+++	DPYSL4 (-44321);JAKMIP3 (+37908)	JAKMIP3
chr14:73246028-73246258	4	3.11E-06	1.04E-02	----	PAPLN (+8646);NUMB (+212387)	PAPLN
chr5:14870484-14870639	3	3.98E-06	1.98E-02	---	ANKH (+1217);OTULIN (+205813)	ANKH
chr11:18411952-18412200	7	4.21E-06	1.31E-02	+++++++	LDHC (-231)	LDHC

**Table 4:** Interpretation of components of the mixed effects model associating baseline MRS, baseline pTau, and longitudinal memory.

Term	Interpretation
intercept	the expected PHC_MEM at baseline visit (time = 0) for the reference covariate levels (sex = male, *APOE4* = 0, mean baseline_pTau, and mean MRS)
baseline_age	changes in baseline PHC_MEM per year of age
PHC_Sex	changes in baseline for females vs males
APOE4	changes in baseline for APOE4 carriers vs noncarriers
PHC_Education	changes in baseline per year of education
MRS	baseline association of MRS with PHC_MEM
time	yearly rate of change in PHC_MEM at mean MRS (i.e., MRS = 0) and mean baseline_pTau (i.e., baseline_pTau = 0)
baseline_pTau	baseline association of pTau with PHC_MEM (at time = 0)
MRS × time	change in the yearly slope per 1-unit higher MRS (positive values indicates higher MRS is associated with slower decline)
baseline_pTau×time	change in the yearly slope per 1-unit higher pTau (negative values indicate faster decline with higher pTau)
MRS × pTau	moderation effect at baseline: change in the baseline pTau-PHC_MEM association per +1 unit higher on MRS (positive values indicate that higher MRS attenuates the harmful effect of pTau on memory at time = 0)
(1|RID)	allows each subject to have their own intercept, accounting for within-person correlation

**Table 5:** Results of the linear mixed-effects model evaluating the association between baseline Methylation Reserve Score (MRS) and longitudinal memory change among 88 MCI participants in ADNI. Shown are fixed-effect estimates. A random intercept was also included to allow each subject to have their own intercept, accounting for within-person correlations across repeated measures.

Term	Estimate	Std.Error	df	t value	Pr(>|t|)	Significance
(Intercept)	−0.253	0.402	81.536	−0.628	5.32E-01	
baseline_age	−0.308	0.064	81.515	−4.837	6.13E-06	***
PHC_Sex	0.117	0.127	80.724	0.917	3.62E-01	
APOE4	−0.480	0.106	81.110	−4.506	2.20E-05	***
PHC_Education	0.041	0.023	81.727	1.794	7.64E-02	
MRS	−0.135	0.071	94.161	−1.903	6.00E-02	
baseline_pTau	−0.138	0.060	96.160	−2.277	2.50E-02	*
time	−0.050	0.008	293.049	−6.212	1.79E-09	***
MRS × time	0.034	0.009	299.310	3.958	9.45E-05	***
baseline_pTau × time	−0.026	0.007	293.607	−3.660	2.99E-04	***
MRS × baseline_pTau	0.217	0.057	84.655	3.837	2.40E-04	***

* *P*-value < 0.05

*** *P*-value < 0.01 *** *P*-value < 0.001

## Data Availability

The ADNI datasets can be accessed from http://adni.loni.usc.edu. The scripts for the analysis performed in this study can be accessed at https://github.com/TransBioInfoLab/ad-cr
